# Business intelligence applied to the consumption of iodinated contrast agents in computed tomography scans

**DOI:** 10.1186/s12911-022-01814-9

**Published:** 2022-03-25

**Authors:** José Rodrigo M. Andrade, Luciano Costa Blomberg

**Affiliations:** Graduate Program in Information Technology and Healthcare Management, Federal University of Health Sciences of Porto Alegre, Porto Alegre, Rio Grande do Sul Brazil

**Keywords:** Business intelligence, Radiology, Diagnostic imaging, Contrast media

## Abstract

**Background:**

The management of the use of iodinated contrast agents (ICA) in the computed tomography (CT) has clinical and financial impacts; however, the approaches in the current research setting have limitations with regard to their exploration of the theme. This work describes the application of the stages of a process of business intelligence (BI), from the formulation of business questions, the building of a research database, and the adaptation of a multidimensional model, to the creation of dashboards to give support to the decision-making process in a hospital. This research aims to apply and document a BI process that provides support to the decision making of managers, so the use of ICA can be better managed, allowing for the identification of situations in which the material was wasted using a study applied to the hospital field.

**Methods:**

An applied exploratory research with a quantitative approach in a database made up by 24 variables and 35,388 records extracted from the RIS (Radiology Information System) that is used by the General Hospital of Porto Alegre—HCPA. The software used, supplied by AGFA Healthcare, were the Qdoc system (version 6.2.0) and the Impax BI (Version 11.1.1) for, respectively, data entry and data exploration. At the end of the process, a total of 48 variables was considered.

**Results:**

The BI process applied allowed for the identification of situations in which ICA was being wasted during the operationalization of the volume/mass ratio of the agent injected in the patient. It also offered the necessary substantiation for the managers to formulate plans, actions, and controls associated to the use of the material. This work made it possible to diminish in 15.65% the total consumption of ICA injected in the patients who underwent the CTAB1 exam (full CT scan of the abdomen), with a projected economy of US$ 10,039.95, for the performance of this exam from 2020 on. The measuring of the impact and the relevance of the process was 99.6% positive, according to the evaluation of the managers.

**Conclusions:**

This research generated clinical and financial benefits for the HCPA, a positive evaluation by the managers and the generation of new knowledge, which can be shared with other public or private health organizations.

**Supplementary Information:**

The online version contains supplementary material available at 10.1186/s12911-022-01814-9.

## Introduction

In Brazil, in 2019, a budget of US$ 23.62 billion was destined for the area of actions in health, according to the transparency portal of the Brazilian General Comptroller's Office [1]. The HCPA, for instance, is a public, general, and teaching hospital which spends approximately US$ 280 million per year to maintain its operation. A recent analysis, provided by the Radiology Service of the HCPA through the management information system (MIS) shows a consumption of materials that reaches US$ 190 thousand per year in the CT unit. 70% of this direct cost expense is represented by the consumption of ICA.

Currently, in the HCPA, the control of the use of materials is carried out by the Storeroom Service, which restocks the material used in each day in the local storage of the Imaging Services. This type of volumetric control of daily use does not allow for a complete managing of the use of the ICA in the CT, nor does it allow for its optimization.

Despite currently having computerized flow systems, the HCPA still requires better tools to support decision making that show its managers, in a fast and safe manner, whether the processes using ICA in the CT are adequate or need to be reviewed and/or improved and/or optimized. In this logic, a better management in the use of the ICA can generate gains from two essential perspectives: one of them related to the clinical effects of the ICA injection (e.g.: nephrotoxicity, fluid overload) [2–4] and another one about the financial point of view with regard to the use of resources.

Previous researches showed that the application of BI in radiology has been generating significant gains to businesses, although new studies on the ICA are still needed. In one of the studies conducted, Nagy et al. [5] described improvements in the waiting time of patients and in the time to issue the results through the application of a BI process which was shown to be significant to aid in the making of more efficient decisions to improve productivity, performance, and the quality in the department. In 2010, Prevedello et al. presented the steps took to create the model-prototype of a BI data repository, showing how it was used to start an investigation of the viability of using new specific KPIs in Radiology. In the same year, the theme "diagnostic time and inspection time" was chosen and discussed by authors Liu, Yu and Li (2010), who built a model to analyze the factors that influenced the number of people attended, the waiting time for the diagnostic and the inspection times for the Radiology sector in three aspects: days of the week, hours, and professional category [5–7].

Another relevant study about the applicability of BI in Radiology was published in 2016, when the authors Jones et al. projected, developed, and implemented the prototype of a BI tool to evaluate the mismatch between capacity and demand. After the BI was analyzed, they carried out a qualitative assessment through interviews with the main interested parties, which reported gains with regard to the ease of information access. More recently, Moriarity, Sigler e Morrison (2020) reported the construction of a data warehouse (DW) in a private Radiology clinic. This approach was elaborated from the point of view of investments in IT and in the team. The authors presented practical applications of analysis and of reports provided to all departments of the organization in the last 5 years, including finances, IT, human resources, billing, coding, clinical operations, and juridical operations. Furthermore, they state that reliable data now inform the aspects of organizational decision making in daily operations, growth, strategic planning, and efficiency [8, 9].

The objective of this research is to apply and document a BI process that provides support to the decision making of managers, so the use of ICA can be better managed, allowing for the identification of situations in which this material was wasted in CT exams in the General Hospital of Porto Alegre.

## Methods

The nature of the tasks involved indicates that the type of research carried out was mainly exploratory. The approach was quantitative, based on the evaluation of the results by the managers and business analysts. The field of application is that of data analysis and support to decision making in the areas of Radiology and Imaging Diagnostics.

The BI process described in this research was structured in three groups of activity, related to:

1. Interests of the managers: the set of activities used to identify the business questions relevant for the organization;
2. Applied technologies: the group of activities involving the use of technologies and strategies for extract, transform and load (ETL), multidimensional modeling, and the generation of information from a set of data from transactional systems of the organization;
3. Discoveries and conclusions: this was the set of activities related to the evaluation and interpretation of data to give support to decision making. The conclusions are obtained in the final activity, from the evaluation of the managers about the process applied.

### Interest of the managers

Initially, the survey about the interests of the managers was carried out, and business questions about consuming ICA in the CT scan were defined. To guide the investigation, a semi-directed interview was elaborated, based on the methodology of Marconi and Lakatos [10], as a way to elucidate the assistance process and document managerial needs (available for access as supplementary material). This instrument, structured in an investigative method made up by six items, included the evaluation of 16 business questions related to ICA consumption. At the end of the process, it was possible to identify more relevant variables and metrics, and a choice was made to formulate a new business question, more closely related to the interests manifested by the interviewee, which guided the development of this work.

### Applied technologies

The selection of interest variables from operational source system was the first activity carried out to seek answers to the formalized business question. The operational source system adopted was the RIS, from the company AGFA Healthcare, through the software Qdoc (version 6.2.0), which uses 11 g Oracle databases, destined to transactional processing (OLTP). This system is employed in the care flow of patients who need to undergo radiology exams in the HCPA, and was the main data source for the selection of the 24 variables of interest, related to the consumption of ICA.

The ETL of the Qdoc for the multidimensional DW model of Impax BI refers to the activity of extracting, transforming, and loading the transactional database into the analytical base. To do so, the Impax BI tool (Version 11.1.1), by AGFA Healthcare, was used. It also adopts Oracle 11 g databases, but is dedicated to analytical processing (OLAP). Among the 31 models available at the DW, the multidimensional request-procedure model was selected, from the subject field of attention provided. This model of Impax BI had already mapped 23 out of the 24 variables of interest selected using the Qdoc. Therefore, an additional variable, which the model lacked, was mapped, and incorporated to the daily routine of ETL.

The multidimensional model was adapted in the DW of the Impax BI, as was the generation of the data cube. This activity consisted in the selection of an additional set of variables of interest that already existed in the Impax BI, in addition to the creation of variables derived from measures calculated in the requisition-procedure model. Considering this new selection, a final set of 48 variables was determined. Filters were applied for the generation of the data cube at Impax BI and the later application of the OLAP. The cube contained 35,388 valid records. It was formed using data about the care provided to 18-year-old or older patients who underwent CT scans using ICA in a 24-month period.

The construction of an investigative dashboard for the use of OLAP operations was a technical activity developed under the permanent advice of the managers, seeking points of view that could facilitate data interpretation. Four charts were created, which were independent, though complementary, in a data dashboard for the iterative manipulation in real response time. The investigative dashboard was conceived to allow one to perform OLAP operations such as slice, dice, drill-dawn, and roll-up, which were applied to the data cube.

### Discoveries and conclusions

Through the consumption and manipulation of data in the investigative dashboard, we sought to answer the business question through managerial analysis, together with the managers. OLAP operations were used in ad hoc investigations, seeking to uncover undesirable events. Considering broader settings, there was an attempt to gradually increase the level of granularity, discussing increasingly specific settings and comparing them to different perspectives with regard to process standardization.

Planning, actions and controls were defined through evidences and results, allowing interested parties to create operational tactics based on the evidence surveyed to plan and act. The BI process continued in the organization after a possibility of improving the standardization of operational processes was found, which involved the definition of an ICA volume for the injection of each patient. A set of changes was applied, mobilizing the interested parties. Two control reports were structured, allowing data to be monitored and enabling comparisons of the scenario in the different stages of the process applied. The changes could be accompanied by the managers through the data sent weekly by the controls generated. To do so, a resource of the Impax BI agent was used, a tool that makes it possible, through its configurations, to automate the sending of reports within a pre-established time frame of interest. Finally, the clinical and financial impacts could be shown and analyzed.

### Statistical analysis

In order to evaluate the statistical significance of the results obtained by reviewing the ICA application protocol, a statistical analysis was performed using the PSPP statistical software. PSPP is an open-source data analysis software intended to be an alternative to IBM SPSS. Two data samples related to the ICA injections for the TCAB1 exams were considered in this work. Statistical analyses were performed, including the Kolmogorov–Smirnov and Wilcoxon Signed Rank tests, adopting a significance level of 5% (*p* < 0.05).

The evaluation of the perception of managers about the applied BI process was carried out at the end of the work, with the HCPA management. To do so, a new semi-directed interview was carried out, based on the methodology of Marconi and Lakatos [10], seeking to quantify the impact and the relevance of the process from the perspective of the interviewee. The results are presented in quantitative form, considering the percentage of the mean of the individual results.

**Fig. 1:**
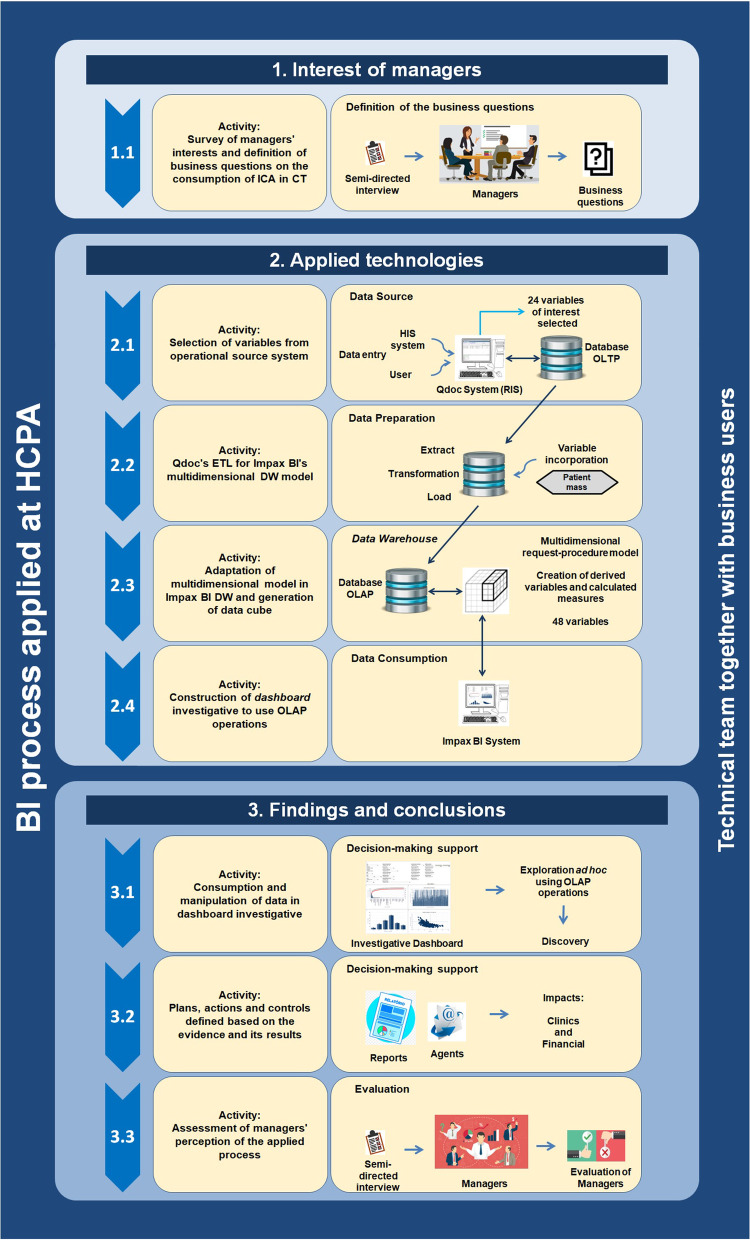
BI process applied in the HCPA (figure created by the authors)

Figure 1 shows a general vision about how the activities are organized within the three groups mentioned.

## Results

This work aimed to apply a BI process in the routine of a large hospital to better manage the consumption of ICA, which is the material with the highest direct expense cost in the CT scan unit. The main interest of managers and business analysts was to search for an opportunity to lower costs, increasing the competitiveness of the organization in the market and generating the greatest possible benefit to the patient.

Survey of the interests of the managers and a definition of business questions about the consumption of ICA in CT scans.

At first, a set of business questions was defined empirically, regarding the evaluation of the ICA volume consumed per scan, and the ratio of volume injected to patient mass. The objective was to identify opportunities to improve assistance processes and reduce expenses of material consumption in the CT scans.

The business questions formulated at first were structured and evaluated through a consultation with the managers of the areas of management, medicine, administration, radiological techniques, and nursing, through a semi-directed interview based on the methodology of Marconi and Lakatos [10]. From this point onwards, we formulated a new business question, more closely related to the interests manifested by the interviewees. As a result, this work was based on the following business question: in which situations is it possible to identify the waste caused by the use of excessive materials?

To better understand the analysis, one must consider:

1. Approach to answer the business question:
  - identifying, in the attention to patients who undergo CT exams with the use of ICA the relations and dependences between (I) the volumes injected and (II) the ratio of volume injected to patient mass, with other variables.
2. Information generated:
  - injected volume, in mL;
  - ratio of injected volume to patient in mL/Kg;
    considered by:
    the patient (patient, mass group, age group, age, mass, and sex);
    exam (specialty, part of the body, name of the exam);
    exam request (request, origin of the attention);
    exam room (exam room, equipment);
    radiology technician (name of the radiology technician);
    nursing technician (name of the nursing technician);
    injection (injection flow, type of injection);
    material (concentration, cost, batch);
    adverse event (extravasation, volume of extravasation, reaction);
    date (date, year, semester, trimester, month, day, day of the week;
    time (time, hour, shift).
  (3) Importance of the information:
    - identifying whether there is a pattern of volume and of volume/mass injected per exam and its dependences, to evidence and investigate situations in which there are signs of material being wasted.

This approach allowed the structuring of data to carry out analysis of the information from different perspectives or dimensions.

### Selection of variables of interest from operational source system

To answer the business question, a search was carried out in the Qdoc for a set of variables that could describe attention with the involvement of ICA injections during CT scans in patients. At first, 24 variables correlated to the care and the consumption of ICA were selected. These were already part of the care flow, and their data was filled in in the Qdoc system.

**Fig. 2:**
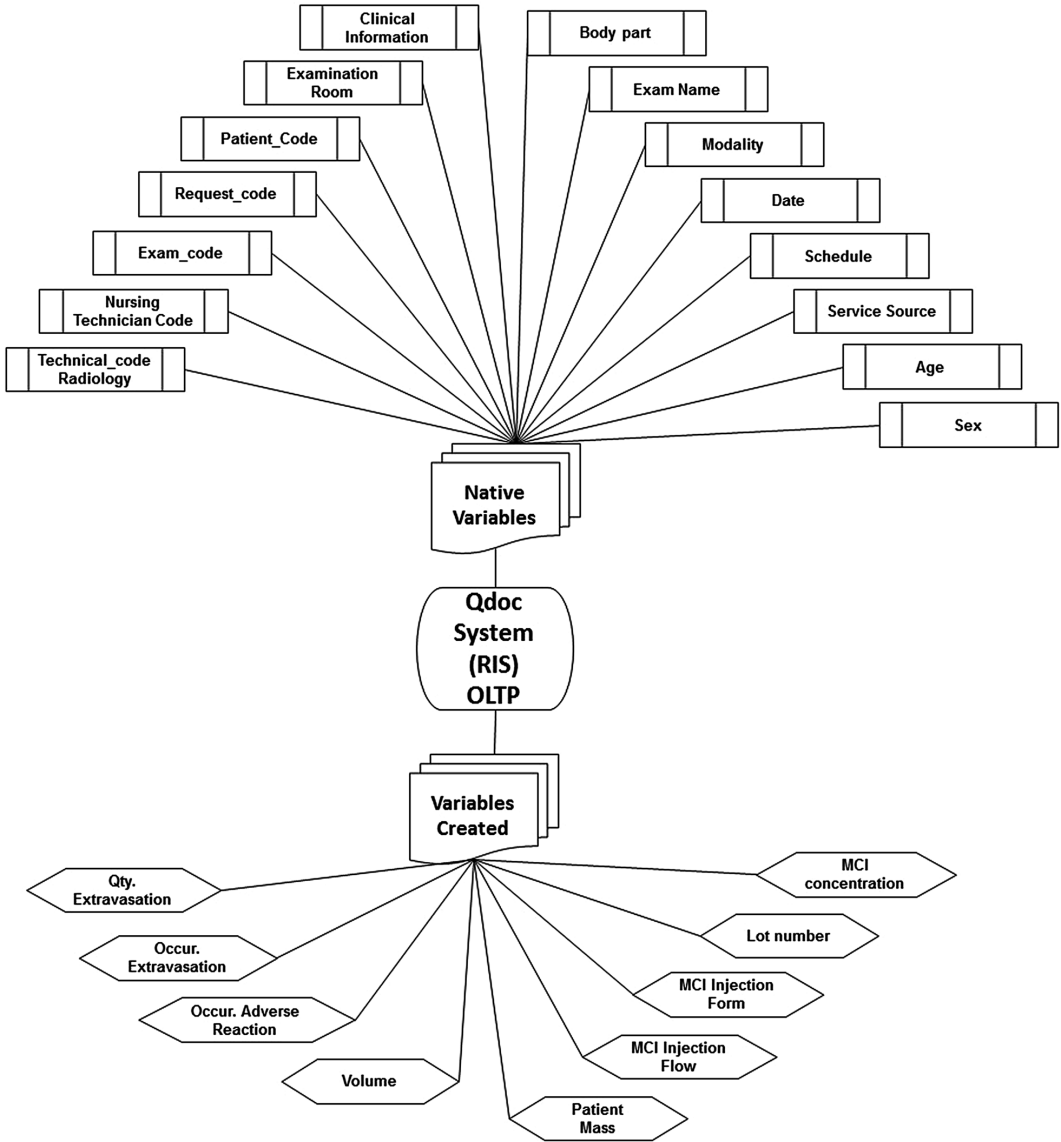
Form of existence of the variables in the Qdoc system: native and created

**Fig. 3:**
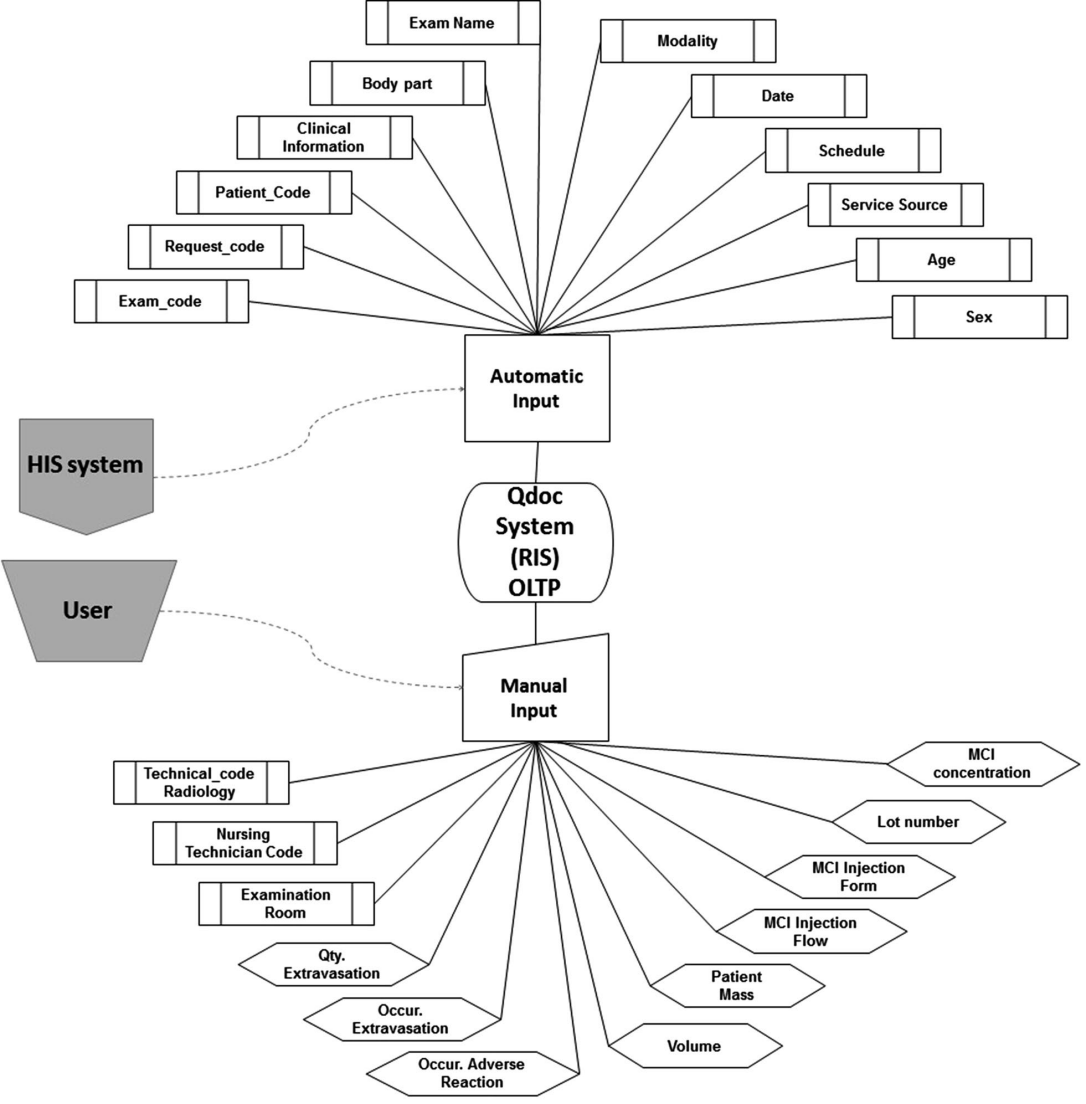
Form of input of variables in the Qdoc system: automatic or manual input

Figures 2 and 3 present the variables of interest of the operacional source system from two perspectives, respectively: form of existence (native or created) and form of input (automatic or manual). Some of these variables are native to the Qdoc system, that is, they exist as a standard inside the software; others were created in the system, by request of the Radiology Service. They were customized to attend to the specific assistance and managerial needs of the institution. Figure 2 shows, in a flowchart, the 24 variables selected from the Qdoc system, highlighting that 15 were native, while 9 were created.

Another criteria to distinguish between the variables is related to the form in which the data is input. It can be automatic, through the integration between the HIS and RIS systems, or manual, through the input of users. As Fig. 3 shows, the data input in the Qdoc system is partly automated, through the process of integration with the hospital system. This takes place when the attention of a new patient starts, which means that a set of data previously registered at the HIS gets transfered to the RIS without the need of filling it in again. A set of 12 variables of interest is inputted in this manner. The other 12 variables depend on the manual input of the Nursing team, as part of the care during the performance of the exam.

### ETL of the Qdoc for the multidimensional model of the Impax BI DW

After the variables of interest were found in the Qdoc system, a multidimensional model of the Impax BI DW was searched, one that could be adapted and used for the objectives of this work. The Impax BI is a market good developed to attend, globally, to the health organizations in the field of Radiology. The AGFA Healthcare company, a multinational in the RIS and PACS industry, adapts Oracle BI, from the Oracle Corporation, developing some multidimensional models and ETL services in its OLTP (RIS and PACS) services for the DW (Oracle).

The request-procedure model was chosen for use in this work since it has a direct correlation with the care effectively offered, since, in the HCPA flow, the requisition of the exam of the patient is sent to the Qdoc system at the moment the patient arrives for its performance.

The Impax BI allows for adaptations, with the mapping of new variables into the preexisting multidimensional models, a service that the AGFA, its supplier, provides. The client is responsible for requesting new mappings according to the interests of the business.

Therefore, from the 24 variables of interest in the Qdoc, 15 had a native mapping for the Impax BI, while 8 were later mapped by request of the organization. Later, the mapping of the variable “mass” was requested, to a total of 9 variables created and considered in the development of this work. It is important to highlight that the new mappings carried out (9 in total) were made viable because of an agreement with the provider of Impax BI, which included the possible addition of up to 15 variables (5 text, 5 number, and 5 boolean variables).

The process of extraction, transformation, and the loading of data from operational database (Qdoc) to the analytical database (Impax BI) is pre-programmed to take place every 24 h, always starting at 12:01 a.m., every day. This inputs all models created with the data generated in the 24 h of the previous day.

### Adaptation of a multidimensional model in the Impax BI DW and generation of the data cube

The multidimensional request-procedure model, available in the Impax BI DW and associated to the field of attention to patients who undergo Radiology exams was adapted to answer to the business question. The first adaptation was the incorporation of the variable of interest patient mass, created in the Qdoc, in the ETL routine, and its mapping in the multidimensional request-procedure model. Therefore, the values filled in to the Qdoc during the attention of the patient started to be extracted from the operational database and to be loaded into the analytical database. With that, as presented in the 1st Quadrant of Table 1, the 24 variables of interest were complete in the same analytical environment.

**Table 1:** Set of variables used in the multidimensional request-procedure model with a description of the variable and of its type of existence in the model

Set	Variable ID	Name of the variable	Description of the variable	Type of existence in the model
1st quadrant: Native and created variables of interest generated by the transaction system and imported into the model	1	Date	Date in which the patient was cared for	NM
	2	Time	Time in which the attention took place	NM
	3	Age (years)	Age of the patient at the day of the attention	NM
	4	Sex	Sex of the patient	NM
	5	Patient mass	Mass of the patient at the day of the attention	CP
	6	Name of the exam	Name of the exam	NM
	7	Part of the body	Part of the body analyzed	NM
	8	Modality	Diagnostic modality (CT scan, magnetic resonance imaging, X-ray, ultrasound scan, etc.)	NM
	9	Origin of attention	Classification of the origin of the attention (outpatient clinic, hospitalization, emergency, external)	NM
	10	Clinical information	Information described by the physician requesting the exam, together with the request and including relevant information on the clinical issues of the patient and the reason why the exam was requested. It accompanies a set of answers to questions of interest of the radiologist, which aim to aid in the medical diagnostic	NM
	11	Exam room	Room in which the exam was conducted (room 1, room 2, room 3, all rooms)	NM
	12	ICA concentration (mg iodine/mL)	ICA concentration (300 mg iodine/mL, 350 mg iodine/mL, all concentrations)	CO
	13	Batch number	Number of the batch of the ICA	CO
	14	Form of ICA injection	Manner in which the ICA was injected (automated with an injector pump, manual with a syringe, all manners)	CO
	15	Injection flow of ICA (mL/s)	Injection flow of the ICA through an endovenous pathway	CO
	16	Occurrence of adverse reactions	Occurrence of adverse reactions	CO
	17	Occurrence of extravasation	Occurrence of extravasation	CO
	18	Amount of extravasation	Amount of extravasation	CO
	19	Pacient_code	Sequential number of the attention within the RIS system	NM
	20	Request_code	Sequential number in the HIS system of exam requests	NM
	21	Exam_code	Identification code of the exam	NM
	22	Radiology Technician_code	Identification code of the Radiology technician	NM
	23	Nursing Technician_code	Identification code of the Nursing technician	NM
	24	ICA volume (mL)	Records of the volume of ICA injected in the patient	CO
2nd quadrant: complementary variables of interest that already existed in the model	25	Year	Year in which attention took place	EM
	26	Month	Name of the month in which attention took place	EM
	27	Day	Number of the day in which the attention took place	EM
	28	Weekday	Weekday in which the attention took place	EM
	29	Semester	Name of the semester in which attention took place	EM
	30	Trimester	Name of the trimester in which attention took place	EM
	31	Hour	Hour in which the attention took place	EM
	32	Equipment	Equipment used to carry out the exam, including a description of the producer and number of channels (Ge 8 channels, Phillips—16 channels, Toshiba—64 channels, all)	EM
	33	Name of the Radiology technician	Name of the Radiology technician that carried out the attention	EM
	34	Name of the Nursing technician	Name of the Nursing technician that carried out the attention	EM
	35	No. of exams	Measure—sum of exams	EM
3rd quadrant: secondary variables and measurements calculated and created in the model	36	Shift	Work shift (morning – 7:00 a.m. to 12:59 p.m, afternoon – 1:00 p.m. to 6:59 p.m, Night – 7:00 p.m to 6:59 a.m., all)	CM
	37	Age group	Age group of the patient at the day of the attention	CM
	38	Mass range	Mass range of the patient at the day of the attention	CM
	39	Specialty	Medical specialty of radiological diagnostic	CM
	40	Exam group	Group of the exam (tomography or angiotomography)	CM
	41	Cost (R$/mL)	Ratio of financial value to ICA volume	MC
	42	Volume per mass (mL/Kg)	Ratio of the volume of ICA injected in the patient to the mass of the patient	MC
	43	Reference values for volume (mL)	Reference values for the volume of ICA to be injected for each combination of exam and weight range of the patient	MC
	44	Excess (mL)	Volume of ICA injected above the volume limit	MC
	45	Cost of the excess (R$)	Cost of the volume of ICA injected above the volume limit	MC
	46	Rate of contrasted patients (%)	Rate of variable 47 to variable 35	MC
	47	No. of contrasted exams	Sum of the exams where variable 12 was filled in	MC
	48	Maximum volume per exam (mL/exam)	Ratio of the sum of the volume of ICA injected in the patient to the sum of the exams carried out	MC

Captions: Acronyms correspond to the form of existence in the multidimensional request-procedure model: NM—native Qdoc variable that previously existed in the model; CO—variable created in the Qdoc and imported into the model by request of the organization; CP—variable created at the Qdoc and imported to into the model at the request of the researchers; EM—variable that already existed in the model; CM—secondary variable created in the model by the researchers; MC—measure calculated and created in the model by the researchers

Since this is a preexisting model, a set of complementary variables of interest, available for use, was developed, according to what is presented in the 2nd quadrant of Table 1. This set of variables is part of those that already existed in the model and was selected to facilitate the analyses.

The second adaptation necessary was the creation, within the multidimensional request-procedure model, of secondary variables and calculated measures. This set of variables, created within the multidimensional model, was selected for use and adaptation and is in the 3rd quadrant of Table 1. For a better perception of the adaptations carried out in the model during the development of the work, the lines of the table related to them are highlighted in a dark tone.

It should be highlighted that the data considered for the development of this work was generated starting in the 1st of July, 2017. The generation of the data cube from the adapted multidimensional request-procedure model was carried out through the application of the following set of filters:

- period from 07/01/2017 to 06/30/2019;
- age of the patient equal or above 18 years of age;
- the modality is CT;
- ICA concentration: is equal to the concentration of 300 mg iodine/mL or 350 mg iodine/mL, that is, the records indicate the use of ICA in the concentrations of interest;
- ICA volume: is not null, that is, the volume of ICA used is filled in;
- ICA volume/mass of the patient: is not null, that is, the ratio of the volume of ICA injected to the mass of the patient is a valid value;
- ICA injection flow: is not null, that is, the injection flow of the ICA used is filled in;
- Mass of the patient: is lower than 300 kg, that is, extremely high values, possible results of mistyping of the ICA volume, are excluded;
- Mass of the patient: is higher than 20 kg, that is, extremely low values, possible results of mistyping of the ICA volume, are excluded;

The cube formed by the filters applied was made up by 35,388 records.

### Construction of an investigative dashboard for the use of OLAP operations

The multidimensional model chosen and the data cube generated lead to a series of viable analyses. Considering the study of situations in which there was waste due to the excessive use of the material, an investigative dashboard was created. It is made up of four charts, making it possible to simultaneously explore all of its variables, forming any desired combination of analysis in real time.

The proposal of the investigative dashboard consolidates four charts that have their presentations altered simultaneously according to the selection, made by the users, of the filters applied to the variables. This structuring is aimed to allow OLAP operations using the data set, enabling slice, dice, drill-dawn, and roll-up procedures.

**Fig. 4:**
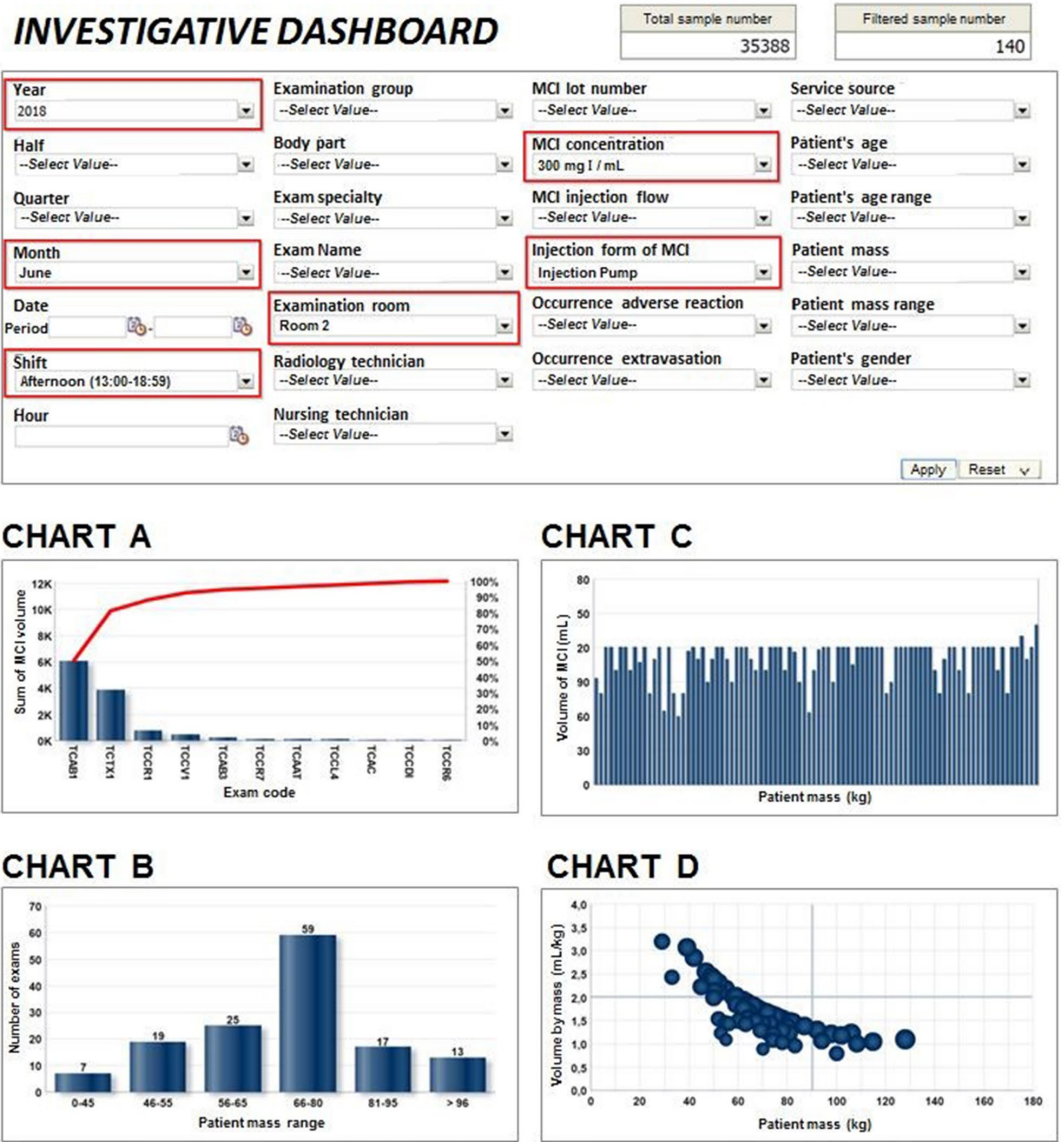
Proposal of an investigative dashboard to answer to the business question using OLAP operations with an example of an analysis from four perspectives

To facilitate these operations in the OLAP cube, a prompt was created, that is, a system component which is a command area for the selection of data, which was made available in the dashboard, with the charts. The prompt allows the user to perform ad hoc explorations according to their interest, applying the same set of selections to all dashboard charts. Figure 4 presents the final dashboard generated, which brings in its wake an investigative proposal which can be adopted by other CT services.

### Consumption and manipulation of data in the investigative dashboard

There was a significant discovery after multiple investigations through ad hoc exploration, starting from the selection of filters in the prompt of the investigative dashboard. This approach considered six perspectives and evaluated varied settings of patient attention. The set of possible combinations explored in this data cube can be noted in Table 2.

**Table 2:** Analysis of the data cube from six perspectives

Perspectives	Selection options
When?	Year: 2017, 2018, 2019, ou all Semester: 1st, 2nd, or all Trimester: 1st, 2nd, 3rd, 4th, or all Month: free choice of month, or all Work shift: morning (7:00 a.m. to 12:59 p.m), afternoon (1:00 p.m. to 6:59 p.m), night (7:00 p.m to 6:59 a.m.), or all)
Where	Room 1, equipped with the 8-channel Ge CT, Room 2, equipped with the 16-channel Philips CT, Room 3, equipped with the 64-channel Toshiba CT, or all rooms
Which?	Individual choice of the code of the exam, or all exam codes
What?	ICA, in a 300 mg Iodine/mL concentration, ICA, in a 350 mg Iodine/mL concentration, or all concentrations
How?	automated ICA injection using an injection pump, manual ICA injection, or all injections
Who?	Individual choice using the code of the radiology technician, or all radiology technicians

**Fig. 5:**
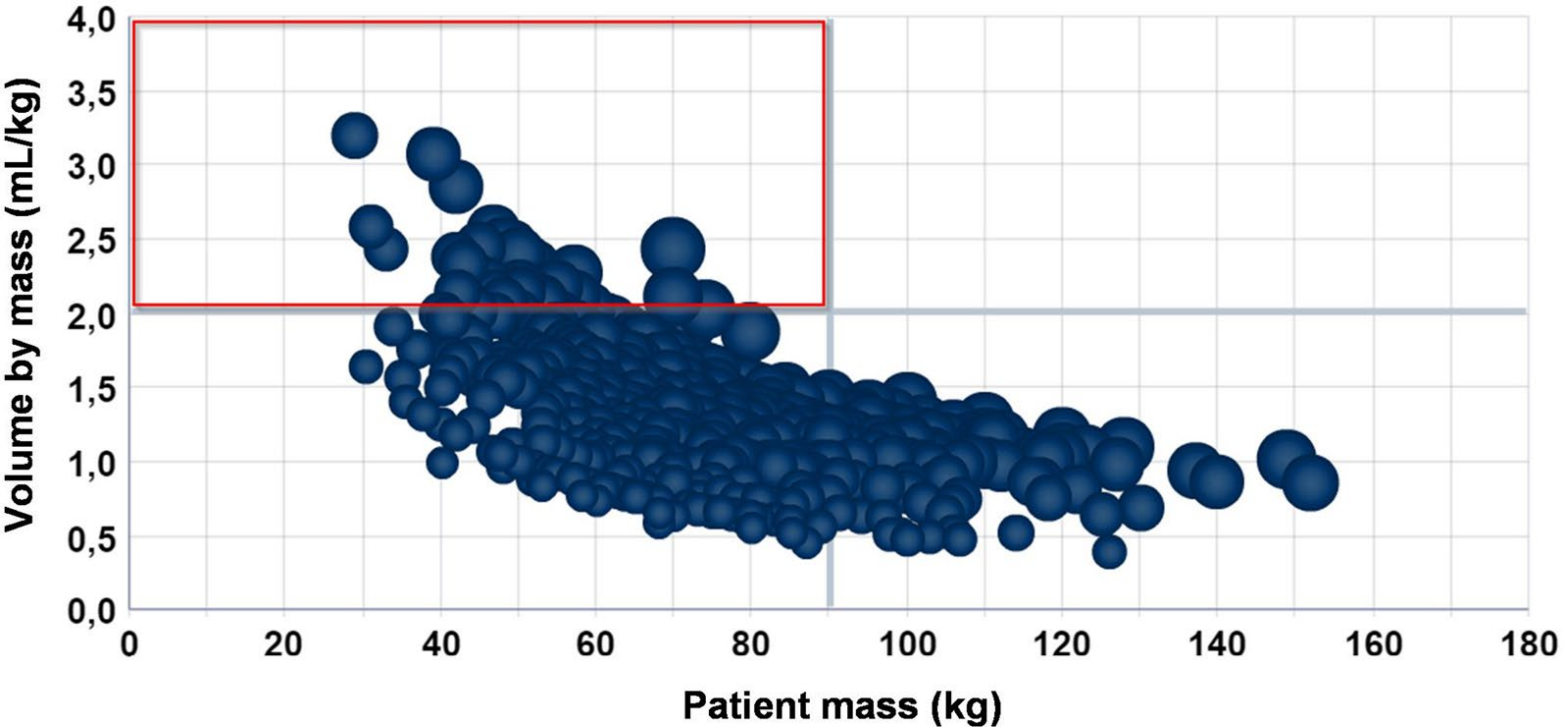
Ratio of ICA volume injected to patient mass in the HCPA Radiology Service in June 2018, with highlights on the quadrant of undesirable situations

Many combinations were tested, always starting from the most general scenario possible and moving towards the most specific one. Figure 5 presents the ratio of the volume to patient mass within a general scenario of the Radiology Service, considering only the year 2018 and the month of June.

The upper-left quadrant of Fig. 5 is highlighted, calling attention to the set of undesirable situations in this broad scenario. The definition of undesirable situation was obtained from a semi-directed interview with the managers. Finding the set of variables that contributes for these situations demands many analyses of the data set, carried out from different perspectives.

**Fig. 6:**
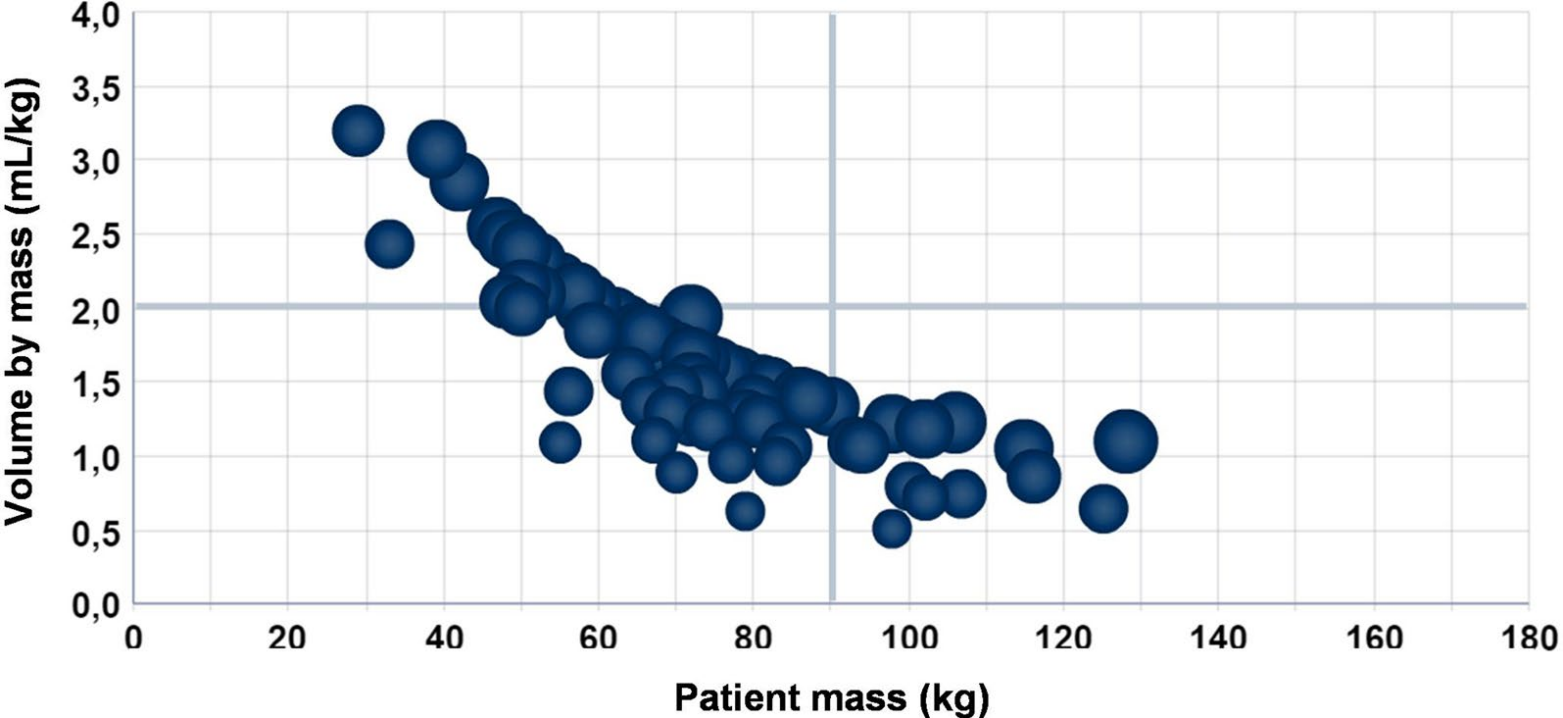
Ratio of ICA volume injected to patient mass in the HCPA Radiology Service in June 2018, by Radiology technician A

**Fig. 7:**
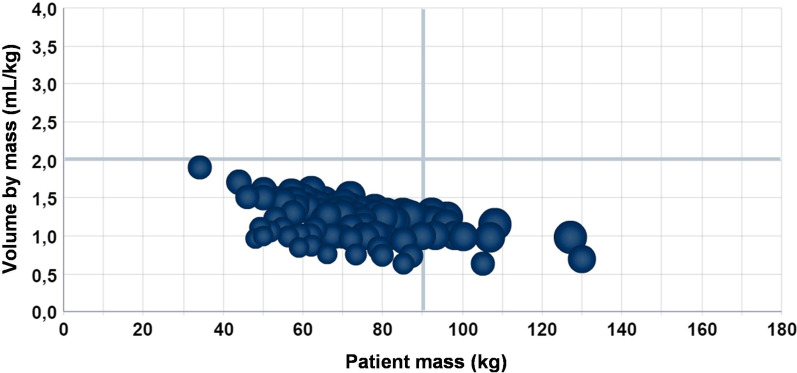
Ratio of ICA volume injected to patient mass in the HCPA Radiology Service in June 2018, by Radiology technician B

The result that better explains the undesired situations was obtained through a variation of the CT operator within this set of data. To explain this observation, Figs. 6 and 7 show the same set of data, filtered to include only patients attended by Radiology technicians A and B, respectively.

After data exploration, it was found that there is an excellent opportunity for the Service to improve the use of ICA volumes in the attention to patients with mass below 65 kg. This finding became visible in Fig. 5, in the highlighted upper-left side of the chart. It could also be noted that the processes of test execution have not been standardized within the Service and with the team, meaning that an effort to this end could make viable a diminution in the consumption of materials, especially for low-mass patients.

It can be noted that the use of simple consultations to databases and reports would not be capable to provide such a fast understanding of the behavior of the data. OLAP technology made it possible to work with many dimensions simultaneously, all of them before analysis. They were considered to be potential contributors for a greater consumption of material. The exploration of data with visual analysis from different perspectives made it possible to identify and prove, through data, whether there was a non-standardized process in the Service, which led to the excessive use of materials.

In this section, there was an attempt to describe the most relevant discovery the investigation made with the data cube to support the decision-making process of the managers.

### Planning, actions and controls defined considering evidences and their results

With the aid of the managers, the investigation continued in the database, seeking to identify the starting point that generated the highest financial and assistance impact. The decision about which exam should be conducted first, as a pilot, was based on data, using a strategy that resorted to the Pareto chart to answer the four defined questions together with the managers. The results are presented in the Table 3.

**Table 3:** Support to decision making in the choice of the pilot exam, using the Pareto chart and considering the data cube

Questions of interest of the managers for decision making, about where to start	Code of the exam				Sum (%)
	TCAB1 (%)	TCTX1 (%)	TCCR1 (%)	OUTROS (%)	
1. Among exams that use the ICA, which one happens the most?	39	25	7	29	100
2. Among exams that use the ICA in a 300 mg iodine/mL, which one happens the most?	47	31	7	15	100
3. Among exams that use the ICA, which one uses the most volume of the material?	42	26	5	27	100
4. Among exams that use the ICA, which one uses the most volume of the material in a 300 mg iodine/mL concentration?	50	31	5	14	100

TCAB1 (full CT scan of the abdomen), TCTX1 (CT scan of the thorax), and TCCR1 (CT scan of the skull/brain)

It can be noted that the TCAB1 is the most representative, considering the four items analyzed. For that reason, it was chosen to be the pilot in the control of the waste of material. This was a strategic decision of the BI process applied towards a greater impact in clinical and financial results. Considering this data-based decision, the planning, together with the managers, was structured in six groups of activity:

1. Reviewing the protocol of ICA injections in the exam selected to be the pilot;
2. Documenting the information thus generated;
3. Generating weekly automated controls;
4. Guiding the team;
5. Accompanying the data;
6. Assessing the clinical and financial impacts.

#### Performance of planned activities and results

The actions that put in practice the planning will be presented with the results of each activity and structured following the same order of organization.

#### Revision of the protocol of ICA injection about the exam chosen to be the pilot

The revision of the protocol of ICA injections for the TCAB1 exam was carried out during multiprofessional weekly meetings involving managers and representatives of the team of Radiology technicians, radiologists, nurses, and physicists. The preexisting guidance available for consultation in the last version of the protocol of exams in the CT unit, revised in January 2019 and referring to the protocol of ICA injections for the TCAB1 exam, was "Intravenous contrast: 1 to 2 mL/kg (up to 150 ml)".

This is an open orientation, leaving the final decision for the Radiology technician at the moment of the execution of the exam. As a result, there was an attempt to find the best association between the volumes injected and the imaging techniques available in the CT Scan, using as a reference the exams from the Service itself, which were evaluated with regard to their quality by the radiologists.

During the review, a strategic change in the protocol was decided. Its aim was to implement guidance with regard to the volume to be used for each concentration and corresponding mass range. Therefore, we tried to achieve a uniform choice of the volume to be injected in the patient and facilitate the decision of the operative team. Finally, a guiding table was formed (Table 4), which is presented below.

**Table 4:** Protocol of ICA injection for TCAB1 exams containing the volume of reference according to mass range and concentration

Mass range of the patient	300 mg iodine/mL	350 mg iodine/mL
Up to 45 kg	54 mL	45 mL
From 46 to 55 kg	70 mL	50 mL
From 56 to 65 kg	80 mL	60 mL
From 66 to 80 kg	90 mL	75 mL
From 81 to 95 kg	100 mL	90 mL
More than 96 kg	110 mL	100 mL

#### Documentation of the information generated

Table 4 shows the new guidance of the Radiology Service for the use of ICA in TCAB1 exams.

#### Generation of weekly automated controls

After the ICA injection protocol was defined for the exam of TCAB1, two control tools were developed using the interest variables of the multidimensional request-procedure model of Impax BI. The controls were generated in the form of a report and programmed to be automatically sent via email to the interested parties every Monday at 7 a.m. This was done using an agent, a resource available in the Impax BI, which contained all data from the exams from last week, with the period of evaluation lasting from Saturday to Friday.

#### Team guidance

After the protocol was reviewed, documenting the information and generation of automated controls, the supervisor of radiologic techniques provided guidance to the team of Radiology technicians in a meeting that involved workers from the three work shifts in a uniform manner, in the first week of the second quarter of December 2019. After this date, the operative team is expected to follow the new ICA injection protocol for all TCAB1 exams. The date in which the new protocol starts to be applied is an important milestone for the comparison of the effectiveness of the actions taken, making it possible to compare the scenario there was before the guidance in Table 4 was implemented to the one after its implementation.

#### Data follow up

Controls started to be sent to the managers and other interested parties who participated in this process in November 9, 2019. The creation of controls before the guidance of the operational team about the new process was important to motivate the team in the search for better results. Furthermore, the period before the guidance of the team about the new process was useful to carry out adjustments in the configuration of reports and data auditing, and from that moment on they started to be delivered with a guarantee that the information contained therein was correct.

After the new ICA injection protocol for TCAB1 exams was in force, there was a diminution in the undesirable situations, above 2 mL/kg, related to the first control. Through the second control, it was possible to verify the standardization of the operational process with a uniformity in the work of the entire team, since the team effectively started to follow the new guidance of ICA injections prescribed by the Service. The strategy of result follow up, with information from the previous week, made it possible for managers to trace actions to intervene, when necessary, in recent occurrences.

#### Evaluation of clinical and financial impacts

**Fig. 8:**
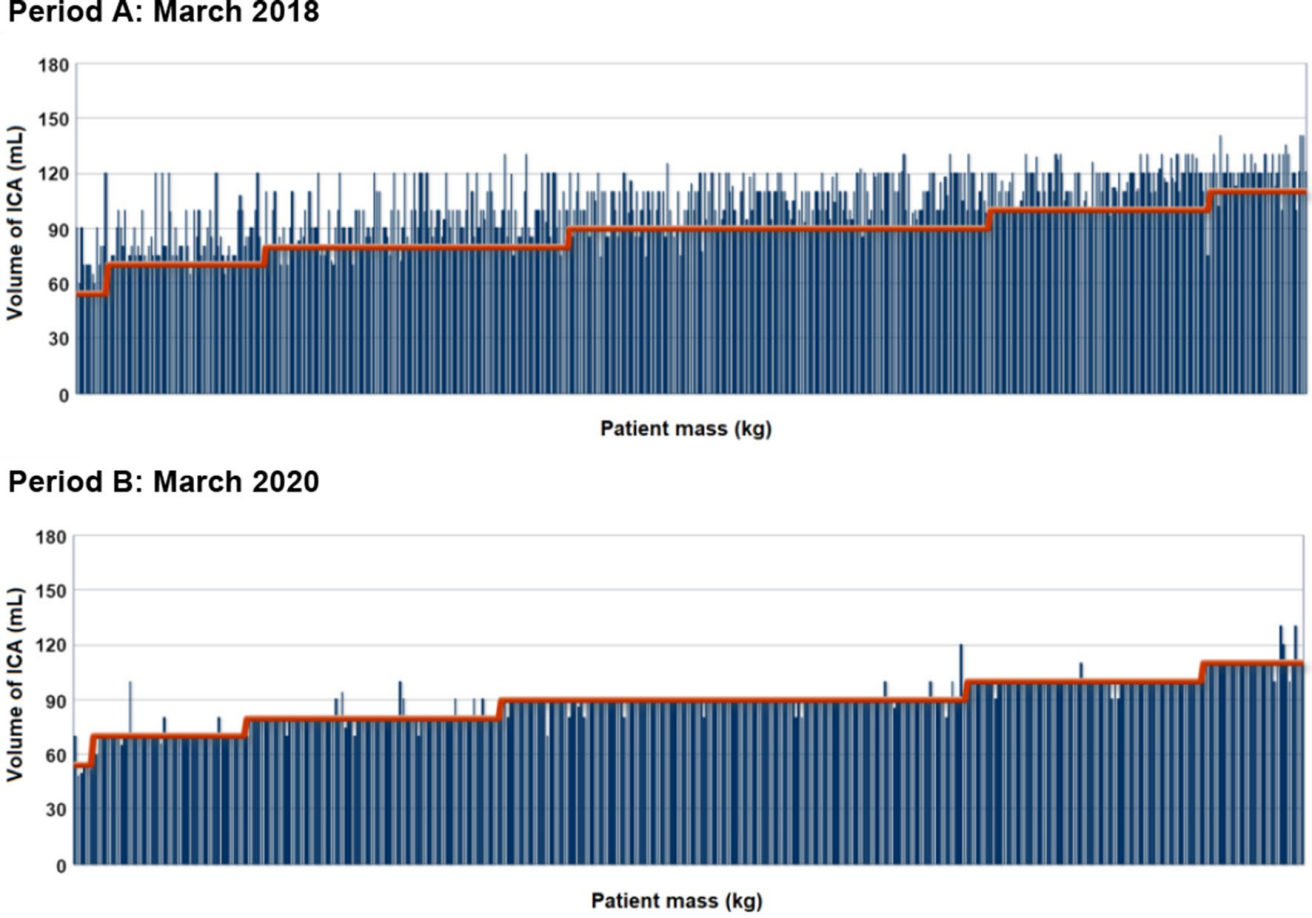
Comparison of data in the periods A and B, respectively corresponding to the scenarios before and after the ICA injection protocol for TCAB1 exams was changed

The BI process applied generated clinical and financial impacts that could be perceived and measured. The clinical impacts are shown in Fig. 8, where data from the periods A and B are compared, respectively corresponding to the scenarios before and after the ICA injection protocol for TCAB1 exams was changed.

In period B, there was a standardization of the ICA injection volumes according to the mass of the patient, and the attention process for the exam analyzed was more uniform than in period A.

**Fig. 9:**
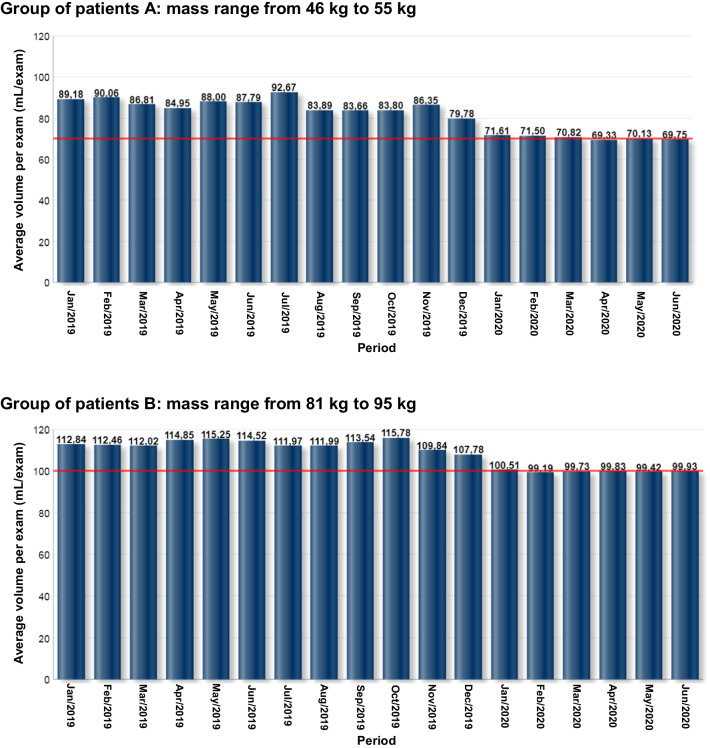
Comparison of the data about the volume of ICA per patient mass range for the TCAB1 exam using ICA in a 300 mg iodine/mL concentration

The clinical gains become more perceptible in the analysis of the mean ICA volume injected per exam in a certain monthly period when the sample varies according to the age group of the patient. Comparative charts presented in Fig. 9 were generated to evidence a diminution in the mean ICA volume injected in the different mass ranges of the patient. The horizontal line in each of the charts indicates the reference value defined in Table 4. The comparison was obtained through the OLAP slice operation, which was applied to the different mass ranges of patients in the data sample selected for analysis. It was found that the ICA volume diminished in all mass ranges, especially for patients below 65 kg.

A projection was also calculated to predict the financial impact of the actions taken after the implantation of the BI process. It adopted, as a reference, the data cube used in the investigative dashboard. Using it, it was possible to evaluate the economic result generated from the definitions of the ICA injection protocol for the TCAB1 exam, due to its clarity in the guidance and standardization of operational processes. To do so, an analysis was carried out that included two distinct 12 month periods from the data cube, as shown in Table 5.

**Table 5:** Assessment of the financial impacts for TCAB1 exams

Information about the measurement	07/01/2017 to 06/30/2018	07/01/2018 to 06/30/2019	Annual mean	Sum (24 months)
Total volume used	719,898 mL	705,699 mL	712,799 mL	1,425,597 mL
Volume that could have been used considering the new ICA injection protocol	602,638 mL	599,850 mL	601,244 mL	1,202,488 mL
Excessive volume used	117,260 mL	105,849 mL	111,555 mL	223,109 mL
Value of the excess	US$ 10,553.40	US$ 9,526.41	US$ 10,039.95	US$ 20,079.81

The value used to calculate the projection is based on the last public notice in the electronic bidding model carried out by the HCPA to acquire ICA. The cost of each mL of 300 mg iodine/mL concentration ICA is of US$ 0.09

### Evaluating the statistical significance of the results

**Fig. 10:**
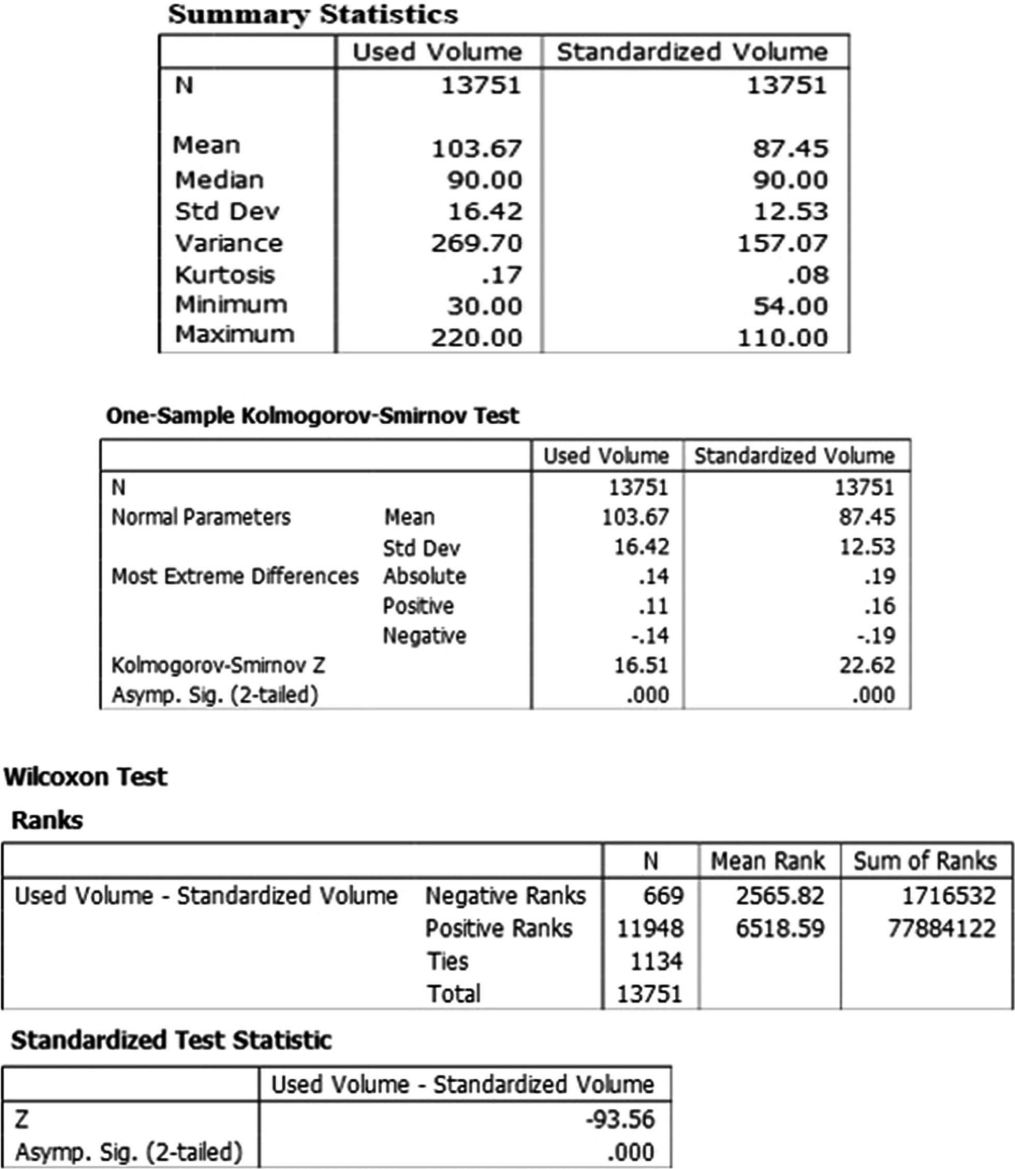
Descreptive analyses

Based on the projection of reduced consumption of ICA for the TCAB1 exams (~ 15,65%), the statistical significance of this difference was found. For this purpose, data referring to 13,751 exams, collected from July/2017 to June/2019 were analyzed. The data were tabulated into two samples dependent on paired data, considering all weight ranges. The first sample referred to the volume of ICA actually used (used volume), while the second was related to the projected consumption for the same exams, considering the review of the ICA application protocol (standardized volume). Figure 10 presents the descriptive statistics.

In order to verify the distribution of sample data, the Kolmogorov–Smirnov statistical test was chosen, assuming as a null hypothesis the existence of normal distribution for each of the analyzed variables.

The normality test showed that the p values (Sig.) for the variables “used volume” and “standardized volume” are both equal to 0.000. Because of the significance < 0.05, this means that the samples’ data are not normally distributed.

Therefore, the statistical analysis continued using non-parametric statistics, indicating the median as a measure of central tendency and assuming the following hypotheses:

H0: The median of the differences between Used and Standardized Volume equals 0.

H1: The median of the differences between Used and Standardized Volume differs 0.

The comparison of medians between pairs of samples was performed using the Wilcoxon test, and since the p value (Sig.) equals 0.000 < α = 0.05 (as seen in the figure below.), the null hypothesis was rejected. Accordingly, it was concluded that the difference between the used and standardized volume is statistically significant.

The difference between the volume used and the standardized volume was of 15.65%, considering only the TCAB1 exam. Therefore, the projection predicted savings of US$ 10,039.95 per year from 2020 on, in the cost of ICA at the HCPA, considering the mean of the 24 previous months used for this pilot exam. This is the cost of the excessive volume projected to be wasted in one year if the BI project had not been implemented. This result, from 2020 on, contributes directly for the sectoral indicator "revenue commited for consumption", which measures the ratio of total financial revenue of the service to all financial values used to pay materials consumed in the corresponding period.

### Assessment of the perception of managers about the process applied

To carry out a quantitative evaluation about the impact and relevance of the BI process applied in the HCPA, a semi-directed interview was elaborated with the management, based on the Marconi and Lakatos [10] methodology, as a way to measure the results achieved.

The interviews were previously scheduled with the same five managers who participated in the first semi-directed interview at the beginning of the work. The meetings were carried out individually through a web conference, which lasted for a mean of one hour. This period was divided in two stages.

In the first stage, the objectives of the work and of the interview were presented to the managers, as well as the research question and the business question of the investigation, its results and its conclusion. In this stage, a report was carried out about the activities developed in the BI process applied, seeking to elucidate the values reached for the business in clinical and financial terms.

In the second stage of the interview, 10 questions were asked, which had been elaborated by the authors of this work. Their answers were in a scale from 0 to 5, and they aimed to quantify the impact and the relevance of each question, where 0 was low and 5 was high. NA meant "does not apply" that is, the dimension was not within the perspective considered by the interviewee. Finally, a mean percentage of the impact and relevance of each question was found. Results are in the Table 6.

**Table 6:** Impact and relevance in the perception of the managers about the BI process as applied in the HCPA

BI process applied in the HCPA	Mean percentage of the impact and relevance in the perception of the managers (%)
Was the result presented satisfactory?	100
Was it important to identify the waste of ICA?	100
Was it important to give support to the decision making process?	96
Did it help in the planning of actions and in the definition of controls from the evidence generated?	100
Did it help in the control of undesirable situations?	100
- ICA injections with values above 2 mL/kg	
- Injections above the reference value indicated in the ICA injection protocol for the TCAB1 exam	
Did it enable a better management of ICA use?	100
Did it generate a satisfactory financial result?	100
Did it generate benefits for the patients?	100
Was it decisive for the follow up of operational routines?	100
Should it continue to be used?	100

One of the interviewees indicated, in the interview, an additional item, which was classified as having 100% of impact and relevance, which was the extinction of the divergences between the teams of Nursing technicians and Radiology technicians about the volume of ICA that should be injected in the patient. Furthermore, the standardization helped the preparation of the ICA injection pump to be faster, since the volume, now, was predefined.

The general score obtained was of 99.6% of impact and relevance in the perception of the interviewees, with regard to the BI process applied to the HCPA. This value was obtained through a mean between all questions answered by the five interviewees.

## Conclusions

Motivated by the constant need of adopting better health management instruments, this work showed that BI technologies can be successfully applied to avoid situations of ICA waste in CT units. With the use of OLAP operations applied to an investigative dashboard to explore a data cube with the managers, it was found in which situations there was an excessive use of the material. The discovery was made when it was found that the Radiology technician influenced the behavior of the curve in the ICA volume/mass ratio injected in the patient. Using this evidence, and based on the data, it was found that the process of defining the volume of ICA injection by the Radiology technician was not standardized.

The strategy, created as part of the BI process, involved a multiprofessional team formed by managers and representatives of the teams of Radiology technicians, radiologists, nurses, and physicists to revise the guidance of the protocol of injection of ICA for the TCAB1 exam, which was the pilot. The open guidance was restructured into a restricted model, according to the mass range of the patient. The creation of two automated controls with structured weekly reports that were sent via email to the interested parties allowed for the data monitoring and the intervention of the managers when needed.

The clinical gains were more expressive for patients with a low mass (below 65 kg); however, the adjustment of the use of the material to the needs of the patients took place for all mass ranges, which now counted on the indication provided by standardized ICA volumes. The financial gains were the consequence of the adjustment carried out considering the clinical needs of diagnostic confirmation in exams that use the ICA. The first exam to be revised by the ICA injection protocol was chosen according to data analysis, with the objective of having a greater impact, and therefore, a decision was made to analyze the TCAB1.

The diminution in the waste of materials due to excessive use brought financial gains to the business. These gains were estimated comparing a historic database of 24 months and the new guidance of the ICA injection protocol for the TCAB1 exam. For this exam, projections indicated a reduction of 15.65% in the use of the material, reflecting in a US$ 10,039.95 diminution in expenses per year, starting from the year 2020.

This work enabled a better managing of the use of ICA, bringing gains from two fundamental perspectives: one of them related to the clinical effects of using lower volumes of contrast, such as a diminution of the nephrotoxicity of the ICA and the lower risk of circulatory overload of the patients, and another from the financial perspective, regarding the efficiency in the use of resources. The perception about the BI process applied to the HCPA was measured through semi-directed interviews responded by the five managers, which obtained a final mean result of 99.6% of impact and relevance, from the point of view of the interviewees.

As a result, the methodological proposal of this work generated an evaluation process capable of producing knowledge with a positive impact, which can be shared with other public or private health organizations. Support was given to the decision-making process of managers, giving base to plans, actions, and controls that favored the business. Its result was an evaluation with a high percentage of impact and relevance, according to the perception of the managers of the application of the BI process.

## Supplementary Information

**Additional file 1:** Results of the semi-directed interview.

## Data Availability

The datasets used and/or analysed during the current study available from the corresponding author on reasonable request.
