# Supplementary material for: Business intelligence applied to the consumption of iodinated contrast agents in computed tomography scans
Source: BMC Med Inform Decis Mak. 2022 Mar 25;22:76. doi: 10.1186/s12911-022-01814-9 (PMC8957133; doi:10.1186/s12911-022-01814-9)
Supplement: Supplementary file 1 — Additional file 1: Results of the semi-directed interview. [file 12911_2022_1814_MOESM1_ESM.docx]

# RESEARCH ARTICLE Open Access

Business Intelligence Applied to the Consumption of Iodinated Contrast Agents in Computed Tomography Scans

José Rodrigo M Andrade^1*^ and Luciano Costa Blomberg^1*^

## **Supplementary material**

## This supplementary material aims to present the result of the semi-directed interview carried out with the managers.

## **Results of the semi-directed interview**

## The semi-directed interview had guiding questions and ample space was given for interviewees to add any comments. The questions and a summary of their responses are presented below:

## **1. Identification of the role of the interviewee in the institution.**

## Five managers of the institution were interviewed, who had the following roles in the company: director (Consulting Office for Operations of Assistance), radiologist (chief of the Computed Tomography Unit), manager (administrative manager of the Radiology Service), nurse (shift leader of the nursing team), and radiology technician (Supervisor of Radiologic Techniques).

## **2. Identifying the person who determines the volume of ICA to be injected in the patient.**

## The interviewees disagreed about who is responsible to determine the volume of ICA to be injected in the patient. According with the interviewees, the decision-making process regarding the volume of ICA per patient was not standardized, structured, and disseminated.

## **3. Identifying whether there is guidance and control.**

## Each interviewee was asked about whether there is a protocol for exams that use the ICA. All interviewees reported that there was.

## They also informed that, as a control tool, the Radiology Service has an indicator called "revenue committed by consumption", and reducing the use of ICA directly impacts this indicator. This indicator is elaborated by the administrative sector by dividing the total revenue of the service by the amount spent to pay for supplies in the same period.

## **4. Identifying the factors that affect ICA consumption. Mapping dimensions.**

## The interviewees were asked about which parameters change the volume of ICA consumption for the exam. The responses found are summarized below:

## S: Yes, N: No, DK: I do not know

| **Yes** | **No** | **I do not know** | **Variables that, when changed, affect ICA consumption** |
| --- | --- | --- | --- |
| 100% | 0% | 0% | The type of exam. (E.g.: thorax) |
| 100% | 0% | 0% | Multiple simultaneous exams (Ex.: skull, thorax, abdomen - in the same prescription) |
| 100% | 0% | 0% | ICA concentration. (E.g.: 300 mg iodine/mL or 350 mg iodine/mL) |
| 100% | 0% | 0% | Injection flow in mL/s |
| 100% | 0% | 0% | Weight of the patient. |
| 100% | 0% | 0% | Clinical information. |
| 100% | 0% | 0% | Patient's kidney function. |
| 80% | 20% | 0% | Venous access. (Ex.: UI intravenous catheter, SR intravenous catheter, central access) |
| 80% | 20% | 0% | Injection using an infusion pump or manual injection with a syringe. |
| 80% | 0% | 20% | Age of the patient. |
| 60% | 40% | 0% | Number of channels of the CT equipment (Eg.: 8, 16, 64 channels) |
| 60% | 40% | 0% | Leakage |
| 60% | 40% | 0% | Adverse reactions. |
| 40% | 60% | 0% | Ionicity. (E.g.: ionic or not ionic) |
| 0% | 100% | 0% | Day of the week or shift in which the exam is carried out. |
| 0% | 100% | 0% | Radiology technician that operates the equipment. |
| 0% | 100% | 0% | Patient's sex. |
| 0% | 100% | 0% | Whether the patient had a recent exam. How many days ago? |
| 0% | 100% | 0% | Use of anesthetic. |
| 0% | 100% | 0% | Origin of the patient. (E.g.: external, outpatient clinic, hospitalized, emergency) |
| 0% | 100% | 0% | Indications of the package insert of the material. Does it change according to the ICA manufacturer? |

## **5. Identifying the relevant metrics.**

## The interviewees were asked about which metrics were relevant, and the following results were found:

| **Relevance** | **Metrics of interest** |
| --- | --- |
| 76% | Volume per weight of the patient [mL/kg] |
| 72% | Costs [R$] |
| 68% | Volume [mL] |
| 68% | Volume per exam [mL/exam] |
| 20% | Cost/exam |
| 16% | Volume/kg per age group |

## **6. Business issues of interest for the managers.**

## We made an attempt to identify what are the business issues of interest for the institution, in regard to the use of contrast in computed tomography, from the point of view of the interviewees and considering the role of each of them in the institution.

| **Relevance** | **Business issue** |
| --- | --- |
| 100% | How much information is missing from the forms that indicate the volume of ICA in CT exams? Can this information be audited to guarantee evidence-based decision making? |
| 80% | Is the volume of ICA used adequate or is it possible to improve? Is it possible to quantify the percentage of adequate uses? |
| 80% | Is the leakage rate under control? |
| 80% | Is the rate of adverse reactions under control? |
| 80% | What is the volume of ICA injected according with the weight of the patient (mL/Kg) for each type of exam, according to the ICA concentration, per month? |
| 80% | Are there changes in the volume (mL) of ICA injected per type of exam, caused by: A. work shift?; B. operating radiology technician?; C. exam room?; D. venous access (Ex.: UI intravenous catheter, SR intravenous catheter, central access); E. others. |
| 80% | Are there changes in the ratio (mL/Kg) of ICA according with the type of exam, caused by: A. work shift?; B. operating radiology technician?; C. exam room?; D. venous access (Ex.: UI intravenous catheter, SR intravenous catheter, central access); E. others. |
| 76% | Are the protocols being followed? Is it possible to control, by creating control guidelines, upper and lower limits for the volume to be injected per exam, and for the mL/Kg ratio per type of exam? |
| 76% | What is the mean volume (mL) of ICA injected according with the type of exam per concentration of ICA per month? |
| 72% | What exams are using a higher-than-expected ICA volume? Is it possible to filter only these exams and search for a correlation between them that explains these anomalies? |
| 68% | What is the ratio of exams with contrast to the total number of exams per month? |
| 68% | What is the total cost (R$) of ICA per month, per concentration? |
| 64% | What is the total volume (mL) used per month, per concentration? |
| 64% | What is the mean value (R$) practiced in the service for the consumption of ICA per type of exam, per concentration of ICA? |
| 60% | What is the rate of use of 300 and 350 contrast for exams that are not angiographies? |
| 56% | What is the rate of use of 300 and 350 contrast for angiographies? |
